# Associations of time-restricted eating with health-related quality of life and sleep in adults: a secondary analysis of two pre-post pilot studies

**DOI:** 10.1186/s40795-020-00402-2

**Published:** 2020-12-17

**Authors:** Dorothea Kesztyüs, Monika Fuchs, Petra Cermak, Tibor Kesztyüs

**Affiliations:** 1 Ulm University Medical Centre, Institute of General Practice, Albert-Einstein-Allee 23, 89081 Ulm, Germany; 2 Georg-August University Medical Centre, Institute of Medical Informatics, Von-Siebold-Str. 3, 37075 Göttingen, Germany

**Keywords:** Time-restricted eating; health-related quality of life, Sleep duration and quality, Employees, Patients, General practitioner, Abdominal obesity, Pilot study

## Abstract

**Background:**

Therapeutic fasting may improve health-related quality of life (HRQoL) and sleep but is not applicable for everyone. Time-restricted eating (TRE) offers a low threshold alternative but research on associations with HRQoL and sleep is rare.

**Methods:**

We conducted a secondary analysis of two pilot studies in a pre-post design, which examined TRE in healthy employees at the Ulm University and in abdominal obese patients in a general practitioners office. Participants reported their HRQoL (EQ-5D visual analogue scale) before and after 3 months of restricting their daily eating to 8–9 h. They kept a diary to protocol timing of first and last meal, sleep quality (analogue scale) and duration. Pearson’s correlation coefficient was applied to test bivariate correlations between continuous variables and linear regression analyses were conducted to identify associated factors with the pre-post differences in HRQoL and the differences in sleep quality.

**Results:**

Ninety-nine participants (aged aged 48.9 ± 1.1, 83.8% female) reached the fasting target of 15–16 h on average on 77.2 ± 18.7% of all recorded days. HRQoL increased by 7.8 ± 12.6 and sleep quality by 9.6 ± 13.9 points, but sleep duration was not extended. Regression analysis revealed mean fasting duration and baseline sleep quality as significant factors associated with changes in HRQoL. Improvements in sleep quality correlated with baseline sleep quality and HRQoL at follow-up but not with fasting. Changes in anthropometry did not correlate with the HRQoL or sleep quality.

**Conclusions:**

TRE correlates with increased HRQoL and sleep quality independent from weight loss. TRE is easily applicable with or without medical supervision. The potential effects of TRE on health and sleep should be further investigated in larger randomized trials.

**Trial registration:**

German Register for Clinical Trials (DRKS), DRKS-ID: DRKS00015057. Registered 4 July 2018.

## Background

Sleep and health-related quality of life (HRQoL) are probably linked, as can be concluded from studies on insomnia [1]. Sleep as well as HRQoL are reported to improve with several days or weeks of therapeutic fasting [2]. This fasting for medical purposes is based on a severe form of caloric restriction to 250–500 kcal/day and is conducted in specialized fasting hospitals or integrative medical departments [3]. Fasting for a shorter period is also used for promoting health and preventing diseases by healthy applicants where it can be monitored by a fasting guide [3]. Therapeutic fasting should not be confused with very low calorie diets or formula diets that restrict energy intake up to 600–800 kcal/day. These diets are also maintained for several days or weeks but are aiming primarily at weight loss and are often subject to the so-called “Yo-Yo Effect”. That is a weight regain unintentionally after returning to the usual diet [4]. Continuous calorie restriction between 20 and 40% less than the average unrestricted food intake, however, has been tested in various trials in different animal species and, to date, seems to be the only non-genetic intervention to increase longevity [5]. In humans some concerns remain about possible negative side effects of calorie restriction [6] and it is understandable that studies in this regard are difficult to conduct. One of the rare studies is the Comprehensive Assessment of Long-term Effects of Reducing Intake of Energy Phase 2 (CALERIE 2) investigation, a randomized controlled trial with 220 healthy non-obese participants. After 2 years of actually 12% calorie restriction, whereby the actual study goal was 25%, significant improvements in sleep and HRQoL were reported [6]. All these aforementioned fasting or calorie restrictive methods are either not feasible without medical or specially trained guides [3], carry the risk of increased weight gain after the end of the dietary phase [4], or place high demands on the adherence of those performing them [6].

Recently, another form of fasting has rapidly gained public interest. Intermittent fasting is the overarching term for a number of nutritional options with temporary calorie restriction. Days with reduced caloric intake alternate with days of normal or even increased intake. The schedule ranges from a daily alternation, over two preferably non-consecutive days, up to only 1 day per week. Intermittent fasting leads to comparable results as continuous calorie restriction in terms of weight loss, reduction in waist or hip circumference, fat mass or fat-free mass lost and ameliorated parameters related to glucose homeostasis [7]. A special form of intermittent fasting is the daily applied so-called time-restricted feeding (TRF), which still bears its origin from animal experiments in its name, but has meanwhile been renamed into “time-restricted eating” by some researchers in this field. TRF or time-restricted eating (TRE) allows ad libitum intake within a given time frame of 3–12 h each day, extending the nightly fast to 12–21 h as a result [8]. In animal studies, TRF was associated with weight loss, reductions in total cholesterol and triglycerides, improvements in glucose metabolism and markers of inflammation [8]. Interestingly, concerning humans, dropout rates in TRE studies were lower than in studies with other intermittent fasting regimes [8]. In a systematic review and meta-analysis of intermittent fasting diets including TRE, a significant decline in body mass index (BMI), fasting glucose, and homeostatic model assessment of insulin resistance (HOMA IR) as compared to controls was reported [9].

To date, only few studies of TRE have investigated sleep. After 16 weeks of TRE with a time frame for eating of 10–11 h, eight healthy overweight adults reported improved sleep [10]. Another study with 23 obese adults who followed an 8 h eating schedule for 12 weeks found no changes in sleep quality or duration [11]. However, we could not identify any TRE study that reported changes in HRQoL. A pilot study in ten sedentary older adults (≥ 65 years) found a tendency to a better quality of life, but this improvement was not statistically significant, possibly due to the small sample size [12].

In 2018 we conducted a pilot study with 63 healthy employees from the Ulm University where our primary outcome was adherence to and feasibility of TRE in adults in a working environment (unpublished material). We found an increase of HRQoL of 5.8 ± 12.4 on a visual analogue scale (EQ-5D VAS), independent of weight loss. In 2019 we conducted another pilot study with 40 abdominal obese patients in a general practitioners (GP) office [13]. Again, our primary goal was to test feasibility and adherence of patients in a GPs office. In the present article, we report results from a secondary data analysis of both studies with regard to changes in sleep quality and duration, and HRQoL after 3 months of TRE with a daily fasting goal of 15–16 h.

## Methods

Both studies were conducted as pilot studies in a pre-post observational design. Details are already reported elsewhere [13]. The primary outcome for both studies was the proportion of days with reaching the fasting goal of ≥15 h out of the total number of days recorded per participant in the diary. According to the study protocol, secondary outcomes were, among others, changes in sleep quality and duration, and HRQoL between baseline and follow-up.

### Recruiting

Participants at the Ulm University were recruited with the support of the occupational health management and by flyers. Exclusion criteria were pre-existing metabolic conditions. Patients at the GP’s office were informed about the study by flyers in the waiting room or were invited by the doctor during a consultation. Exclusion criteria were insulin dependent diabetes or any other disease for which fasting is contraindicated [3]. Finally, 63 participants at the Ulm University and 40 participants at the GP’s office were included in the studies.

### Intervention

Participants in both studies were asked to limit their daily food intake to 8–9 h and subsequently extend their nightly fasting period to 15–16 h. The duration of the intervention was 3 months. At baseline, participants had an introductory conversation with the principal investigator or the physician to clarify possible questions and problems in advance, and were given an information brochure. In addition, all participants were offered to contact the respective study centre at any time if they had questions or problems.

### Data assessment

Baseline assessment comprised a questionnaire to collect data on lifestyle, health behaviour and HRQoL (EQ-5D VAS) [14], and anthropometric measurements of waist, height, and weight (for details see [13]). All participants were given a diary to record the times of their first and their last meal, and the quality and duration of their sleep. The latter was assessed on a visual analogue scale ranging from 0 (worst possible sleep quality) to 100 (best possible sleep quality). The waist-to-height ratio (WHtR) was calculated by the division of waist by height in centimetre, abdominal obesity was then defined as WHtR ≥0.5, as recommended by the literature [15]. Body weight in kilogram was divided by height in meters squared to determine body mass index (BMI), and subsequently categorized into overweight (≥ 25) and obesity (≥ 30).

After 3 months, follow-up measurements were performed in the same manner, with some additional items in the questionnaire regarding the individual experience and attitudes towards TRE.

### Statistical analysis

Baseline characteristics are reported descriptively for each study group and for both groups combined. Differences between groups were tested by applying t-test, Welch’s t-test or Mann Whitney U test according to distribution and heterogeneity in variance for continuous data, and Fisher’s exact test for categorical data.

Follow-up data, and computational differences between baseline and follow-up data, presented as the respective Δ, were treated the same way. Pre and post comparisons for both groups taken together were determined by the Wilcoxon signed-rank test for related samples.

For each participant, mean values and standard deviations were calculated for the data from the diaries. Time of first meal and time of last meal were utilized to determine the duration of the fasting and the eating phase. For all days recorded, the percentage of days with fasting target reached was calculated. Differences between groups were tested as described above.

To assess differences between sleep duration and quality at the beginning and at the end of the TRE intervention period, mean values were calculated for the first 10% and the last 10% of data (or days), respectively. Subsequently the differences between the first and the last 10% of the data were calculated as the respective Δ. They are reported together with the average number of days recorded per group and for the whole group.

Pearson’s correlation coefficient was applied to test bivariate correlations between continuous variables.

Linear regression analyses were conducted for the pre-post differences in HRQoL and the differences in sleep quality between the first 10% and the last 10% of days recorded. Potential factors that might correlate with the HRQoL or sleep quality were identified and, together with variables that differed at baseline between both groups, tested in a stepwise backward elimination. Sex, age, baseline values of HRQoL, the sleep quality and sleep duration on the first 10% of reported days, mean duration of fasting, percentage of fasting target reached, and finally group membership were considered as potential associated factors. Anthropometric measures represented both, potential associated factors and differences between groups at baseline. Therefore, weight, waist circumference, BMI, WHtR, overweight, obesity, abdominal obesity as well as the respective Δ between pre and post measures of the continuous variables were considered in the regression analysis. All assumptions of linear regression (linear relationship, multivariate normality, multicollinearity, auto-correlation, homoscedasticity) were examined.

The significance level for two-sided tests was set at α = 0.05. All statistical analyses were carried out by using the statistical software packages IBM SPSS Statistics for Windows, Version 25.0. (IBM Corp., Armonk, NY, USA).

## Results

There were two dropouts in each study group. Reasons were personal overload, illness, occupational stress, and in one case unknown. Finally, data from 99 participants were available.

The baseline characteristics of the participants are shown in Table 1.

**Table 1:** Baseline characteristics of participants in the TRE pilot studies 2018/19

	University (***n*** = 61)	GP (***n*** = 38)	Total (***n*** = 99)
Age, years M (SD)	48.4 (10.3)	49.7 (11.8)	48.9 (1.1)
Female, *n* (%)	53 (86.9)	30 (78.9)	83 (83.8)
Weight, kg M (SD)	**73.9 (14.3)**^**1**^	**87.9 (21.3)**	79.3 (1.9)
Waist circumference, cm M (SD)	**89.1 (12.2)**^**2**^	**106.3 (13.3)**	95.7 (15.1)
BMI, kg/m^2^ M (SD)	**26.1 (4.7)**^**2**^	**31.2 (5.9)**	28.0 (5.7)
WHtR, M (SD)	**0.53 (0.07)**^**2**^	**0.64 (0.07)**	0.57 (0.09)
Overweight, *n* (%)	18 (29.5)	12 (31.6)	30 (30.3)
Obesity, *n* (%)	**11 (18.0)**^**2**^	**21 (55.3)**	32 (32.3)
Abdominal obesity, *n* (%)	**36 (59.0)**^**2**^	**38 (100.0)**	74 (74.7)
HRQoL, M (SD)^a^	**75.1 (13.2)**^**3**^	**68.3 (13.7)**	72.2 (13.8)
Daily eating time, h M (SD)^b^	12.4 (1.8)	12.3 (1.2)	64.6 (15.6)

NOTE. *TRE* Time-restricted eating, *GP* General practitioner, *M* Mean, *SD* Standard deviation, *kg* Kilogram, *cm* Centimetre, *h* Hours, *BMI* Body mass index, *WHtR* Waist-to-height ratio, *HRQoL* Health-related quality of life; ^a^10 missing values; ^b^1 missing value; significant values are bold; *p*-values of group differences: ^1^ *p* = 0.001; ^2^ *p* < 0.001; ^3^ *p* = 0.024

Differences between both study groups were significant for weight, waist circumference, BMI, WHtR, obesity, and abdominal obesity where participants from the Ulm University had lower values. On the other hand, participants from the GP’s office showed lower baseline values in HRQoL. At baseline, HRQoL and sleep quality showed a positive correlation (*r* = 0.254, *n* = 89, *p* = 0.016).

### Follow-up results

At follow-up, significant differences between groups have persisted for weight, waist circumference, BMI, WHtR, obesity and abdominal obesity, whereas the difference in HRQoL has been balanced out during the course of the intervention. Participants in the GP’s office experienced greater reductions in waist circumference and WHtR than those at the University. Regarding the whole group, follow-up values differed from baseline values for weight, waist circumference, BMI, WHtR and HRQoL. While anthropometric measures significantly declined, HRQoL significantly increased by 7.8 ± 12.6 (*p* < 0.001) points for the whole group. Details are depicted in Table 2.

**Table 2:** Follow-Up results of participants in the TRE pilot studies 2018/19

	University (***n*** = 61)	GP (***n*** = 38)	Total (***n*** = 99)
Weight, kg M (SD)	**72.6 (14.1)**^**1**^	**86.1 (21.8)**	77.8 (18.6)
Waist circumference, cm M (SD)	**87.4 (12.0)**^**2**^	**100.7 (13.9)**	92.5 (14.3)
BMI, kg/m^2^ M (SD)	**25.6 (4.6)**^**2**^	**30.5 (6.0)**	27.5 (5.7)
WHtR, M (SD)	**0.52 (0.07)**^**2**^	**0.60 (0.07)**	0.55 (0.08)
Overweight, *n* (%)	18 (29.5)	8 (21.1)	26 (26.3)
Obesity, *n* (%)	**11 (18.0)**^**3**^	**20 (52.6)**	31 (31.3)
Abdominal obesity, *n* (%)	**36 (59.0)**^**2**^	**35 (92.1)**	71 (71.7)
HRQoL, M (SD)^a^	80.2 (11.0)	78.6 (11.8)	79.6 (11.3)
Δ Weight, kg M (SD)	−1.3 (2.3)	−1.8 (2.6)	**−1.5 (2.4)**^**a1**^
Δ Waist circumference, cm M (SD)	**−1.7 (3.2)**^**2**^	**−5.5 (2.9)**	**−3.2 (3.6)**^**a1**^
Δ BMI, M (SD)	−0.4 (0.8)	−0.7 (0.9)	**−0.5 (0.9)**^**a1**^
Δ WHtR, M (SD)	**−0.01 (0.02)**^**2**^	**− 0.03 (0.02)**	**− 0.02 (0.02)**^**a1**^
Δ HRQoL, M (SD)^b^	5.84 (12.4)	10.3 (12.6)	**7.8 (12.6)**^**a1**^

NOTE. *TRE* Time-restricted eating, *GP* General practitioner, *M* Mean, *SD* Standard deviationm, *kg* Kilogram, *cm* Centimeter, *h* Hours, *BMI* Body mass index, *WHtR* Waist-to-height ratio, *HRQoL* Health-related quality of life; ^a^2 missing values; ^b^10 missing value; Δ = differences between baseline and follow-up; significant values are bold; *p*-values of group differences: ^1^ *p* = 0.002; ^2^ *p* < 0.001; ^3^ *p* = 0.001; *p-*values of differences between baseline and follow-up: ^a1^ *p* < 0.001

HRQoL and sleep quality were positively correlated at follow-up (*r* = 0.513, *n* = 97, *p* < 0.001), while Δ HRQoL and Δ sleep quality showed no significant correlation (*r* = 0.071, *n* = 88, *p* = 0.509).

### Results from the diaries

There were no significant differences between groups regarding sleep duration and quality. The timing of the first meal was equal in both groups, contrary to the timing of the last meal, where the participants from the University ate about 40 min later, so that the period of food intake was extended and the fasting phase shortened, accordingly. Participants from the University reached the fasting target of ≥15 h on 72.0 ± 19.0% of all recorded days, those from the GP’s office on 85.4 ± 15.2% of all recorded days. See Table 3 for more details.

**Table 3:** Diaries of participants in the TRE pilot studies 2018/19

	University (***n*** = 61)	GP (***n*** = 38)	Total (***n*** = 99)
Sleep duration, h M (SD)	7.4 (0.6)	7.4 (0.7)	7.4 (0.6)
Sleep quality, M (SD)	73.4 (14.0)	68.6 (15.0)	71.6 (14.5)
Time of first meal, M (SD)	10.4 (1.3)	10.4 (1.8)	10.4 (1.5)
Time of last meal, M (SD)	**18.7 (1.0)**^**1**^	**18.0 (1.7)**	18.5 (1.3)
Eating phase, h M (SD)	**8.4 (0.9)**^**2**^	**7.7 (0.8)**	8.1 (0.9)
Fasting phase, h M (SD)	**15.6 (0.9)**^**2**^	**16.3 (0.8)**	15.9 (0.9)
Fasting target reached, % M (SD)	**72.0 (19.0)**^**3**^	**85.4 (15.2)**	77.2 (18.7)

NOTE. *TRE* Time-restricted eating, *GP* General practitioner, *M* Mean, *SD* Standard deviation, *h* Hours; significant values are bold; *p*-values of group differences

^1^ *p* = 0.019; ^2^ *p* = 0.001; ^3^ *p* < 0.001

### Sleep duration and quality

Participants recorded the quality and duration of their sleep on average on 89 days during the TRE intervention. A statistically significant difference regarding the number of days recorded between both groups occurred, but this difference of 0.3 days is not meaningful for the further evaluation. Sleep duration did not change between the first 10% and the last 10% of days recorded. However, sleep quality changed significantly by 9.6 ± 13.9 (*p* < 0.001) points on the visual analogue scale, with no significant difference between groups. More information is given in Table 4.

**Table 4:** Sleep duration and quality, first 10% values, last 10% values and differences in the TRE pilot studies 2018/19

	University (***n*** = 61)	GP (***n*** = 38)	Total (***n*** = 99)
Number of recorded days, M (SD)	**88.8 (5.5)**^**1**^	**88.5 (9.9)**	88.7 (7.4)
Sleep duration first 10%, M (SD)	7.4 (0.5)	7.5 (0.9)	7.5 (0.7)
Sleep duration last 10%, M (SD)	7.3 (0.7)	7.5 (0.8)	7.4 (0.8)
Δ Sleep duration, M (SD)	0.1 (0.6)	0.1 (0.8)	0.1 (0.7)
Sleep quality first 10%, M (SD)	66.2 (16.0)	62.0 (14.8)	64.6 (15.6)
Sleep quality last 10%, M (SD)	75.4 (16.0)	72.2 (16.1)	74.2 (16.2)
Δ Sleep quality, M (SD)	9.3 (14.1)	10.2 (13.7)	**9.6 (13.9)**^**a1**^

**NOTE.** *TRE* Time-restricted eating, *GP* General practitioner, *M* Mean, *SD* Standard deviation; significant values are bold; *p*-values of group differences: ^1^ *p* = 0.033; *p*-values of differences between baseline and follow-up: ^a1^*p* < 0.001

**Fig. 1:**
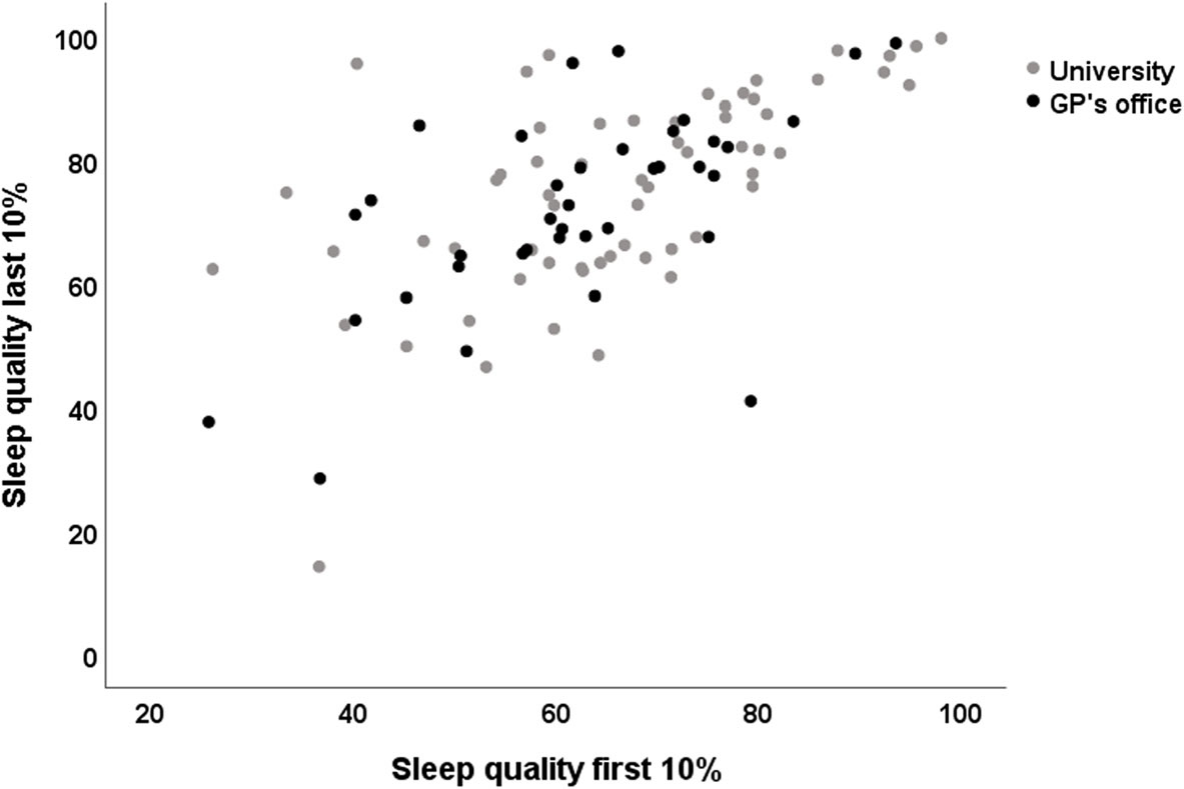
Self-reported sleep quality at the beginning and at the end of the intervention phase. Scatterplot of the sleep quality of the 10% first days (x-axis) and the 10% last days (y-axis) reported in the diaries of participants (*n* = 99) in the TRE pilot studies (2018/19)

The only correlated factors on changes in sleep quality that could be identified in the linear regression analysis were the sleep quality on the first 10% of reported days, and the HRQoL measured at follow-up. Changes in the quality of sleep between the first 10% and the last 10% of reported days are visualized in Fig. 1.

### Linear regression analysis for HRQoL

The average difference in HRQoL from baseline to follow-up was 7.8 ± 12.6 points on the visual analogue scale (EQ-5D VAS), ranging from zero, worst imaginable quality, to 100, best imaginable quality. We analyzed factors with potential associations with this difference in a linear regression model. No anthropometric factor correlated with the outcome; neither sleep quality nor sleep duration showed an association. Age and sex also did not correlate with the increasing HRQoL. Regression analysis revealed significant associations of the baseline HRQoL values, the mean fasting time, and the initial sleep quality, indicating positive correlations.. Results are presented in Table 5.

**Table 5:** Results of the linear regression analysis for the change in HRQoL after 3 months of TRE in healthy participants at the Ulm University (2018) and abdominal obese participants in the GP’s office (2019), *n* = 87

Covariate	***B (SE)***	***ß***	***p-Value***
HRQoL baseline	−0.64 (0.07)	−0.71	< 0.001
Mean fasting time	2.43 (1.16)	0.17	0.039
Sleep quality first 10%	0.27 (0.07)	0.32	< 0.001
Study arm GP’s office	−0.15 (2.14)	−0.006	0.946
Adjusted *R*^2^	0.49		

**Note.** *TRE* Time-restricted eating, *GP* General practitioner; *B* regression coefficient, *SE* Standard error, *β* = standardized regression coefficient

The present linear regression fulfilled all assumptions. The R^2^ for the overall model was 0.51 (adjusted R^2^ = 0.49), indicative for a high goodness-of-fit according to Cohen. HRQoL, mean fasting time and baseline sleep quality were able to statistically significant predict changes in HRQoL, F (4, 83) = 21.57, *p* < 0.001. Membership in a study arm had no significant influence on the outcome.

## Discussion

Three months of TRE correlated with changes in HRQoL by 7.8 ± 12.6 and sleep quality by 9.6 ± 13.9 points on visual analogue scales ranging from zero (worst quality) to 100 (best quality), but no extension of sleep duration was observed in two pilot studies with 99 participants. Interestingly, these parallel changes were independent from each other and independent from weight loss. Differences in HRQoL depended on mean fasting duration, baseline quality of sleep and HRQoL. It seems the longer the nightly fast, the better the HRQoL. Changes in sleep quality depended on baseline sleep quality and were correlated with HRQoL at follow-up. Sleep quality and HRQoL were positively correlated at baseline and follow-up, respectively. TRE was well accepted by participants with little side effects, which ameliorated in the course of the study [13].

HRQoL as a patient reported outcome is substantial for decisions about new treatments in the National Institute for Health and Care Excellence (NICE) of the National Health Service of the United Kingdom [16]. The preferred instrument for the assessment of self-rated HRQoL is the EQ-5D, from which the visual analogue scale (VAS) was taken for the TRE examinations presented here. The VAS is a validated instrument for assessing self-rated health [17]. The association between self-rated health and objective health status has been examined in a large population-based study indicating a consistent relationship [18]. HRQoL is a so-called “patient-reported outcome” (PRO) which is defined by the US Food and Drug Administration (FDA) as any statement about a patient’s health status made directly by the patient himself, without interpretation by a clinician or anyone else [19]. PROs are important because they make it possible to determine the effects of a treatment directly experienced by the patient beyond measurable clinical parameters. The changes in HRQoL associated with the TRE intervention was significant and independent from weight loss or reductions in abdominal obesity. With respect to the initial values, participants in the GPs office increased their HRQoL by 15.1%, those at the Ulm University by 7.8%. There is little information from research in the field of fasting concerning HRQoL, so this is one of the rare studies to add information to this topic.

Research on TRE and sleep is very rare. One study reported a significant increase in sleep satisfaction within 16 weeks of restricting daily eating duration to 10–11 h in eight healthy overweight individuals [10]. Another trial found good sleep quality throughout a 12 week intervention with a restricted eating time of 8 hours daily in 23 obese adults [11]. Participants in our study reported improved quality of sleep from initially 64.6 ± 15.6 to 74.2 ± 16.2 on a visual analogue scale ranging from zero to 100. Hence, restricting the time frame of eating seems to be associated with quality of sleep, but not, at least in our examination, sleep duration.

Therapeutic fasting may improve sleep [2], but some religious forms like Ramadan fasting may even reduce sleep duration and increase daytime sleepiness [20]. Sleep is of essential importance for our physical and mental health and poor sleep over a longer period increases the risk of health problems [21]. Sleep debt was identified to have an overall negative impact on metabolic and endocrine function many years ago [22], and was associated with an increased risk of diabetes in middle-aged men [23]. Newer studies indicate that sleep loss nowadays is, in parallel with the pandemic prevalence of obesity and type 2 diabetes, a specific problem of modern societies [24]. Interestingly, shift work, known for its disruption of circadian rhythmicity, was independently associated with poor sleeping quality and an increased risk of hypertension and type 2 diabetes in more than 25 thousand retired Chinese workers [25]. Not only the light/dark cycle but the feeding/fasting cycle affects the circadian system of the human metabolism and TRE may help to restore circadian rhythmicity as it mimics the way men ate ten thousands of years throughout the evolution [26].

TRE is primarily investigated in terms of its benefits for weight reduction and metabolic improvement. The participants in our study were able to reduce both their weight and waist circumference, although these reductions were more pronounced in patients in the family practice. TRE may be less suitable as a temporary measure of weight loss since this happens slowly, but as a permanent lifestyle change for longer-term normalization of the weight and metabolism. Beyond weight loss and improved quality of life and sleep, TRE may have further positive influences on health psychological determinants such as wellbeing, happiness and self-efficacy expectation (unpublished data).

### Strengths and limitations

Firstly, in each study group only two participants discontinued the intervention, which was lower than expected. Overall, with the exception of the baseline HRQoL of participants at the Ulm University, there were no missing values from questionnaires or anthropometric examinations. One strength of this secondary analysis lies within the density of data regarding sleep. Participants evaluated the duration and quality of their sleep on every single day for the night before. The adherence of the participants was excellent and only few reported difficulties in implementing TRE.

Due to the pilot character of the studies, no control groups were included. Unfortunately, for the study group at the Ulm University the VAS for the assessment of the HRQoL at baseline was printed on the last backside of the questionnaire and was therefore overlooked by 10 participants. Furthermore, not all participants recorded the full number of days in their diary during the 3 months of the intervention. Men are underrepresented in both groups, a fact that has already been noted in other studies. For instance, male participation in programs for chronic disease self-management education remained low [27].

Bias occurred from several sources in this research. Firstly, selection bias is most obvious because participants either decided themselves to take part or were invited by the doctor. Blinding of staff and participants was not possible due to the study design and the kind of intervention, leading to performance and detection bias. Thanks to single-digit numbers of dropouts, attrition bias regarding missing outcome data was low. Social desirability as well as recall bias may have arisen with regard to the data from the diaries. It should be noted that the sleep quality of the 10% first days may have been compromised by the change in meal timing and therefore may have been reduced, possibly leading to an overestimation of the effect of TRE on sleep quality. Furthermore, all data on meal timing and sleep quality were self-reported. Finally, gender bias is very strong with a ratio of 8:2 in favour of women.

In summary, the reported results are exploratory and should be interpreted with caution.

## Conclusion

TRE offers a low threshold intervention with the potential to increase the health-related quality of life and sleep quality in the adult population. It is suitable for primary care as well as for self-administration. The results give reason to implement further preferably randomized controlled trials to gain deeper insights into the benefits of a non-pharmaceutical intervention for better sleep and quality of life.

## Data Availability

The datasets generated and analysed during the current study are not publicly available due to reasons of data protection (German Data Protection Act) but are available from the responsible data manager, Prof. Dr. Tibor Kesztyüs, on reasonable request.

## Abbreviations

BMI: Body mass index
CALERIE: Comprehensive Assessment of Long-term Effects of Reducing Intake of Energy
HOMA IR: Homeostatic model assessment of insulin resistance
HRQoL: Health-related quality of life
TRE: Time-restricted eating (used for human studies)
TRF: Time-restricted feeding (used for animal studies)
EQ-5D VAS: European Quality of Life 5 Dimensions Visual Analogue Scale
WC: Waist circumference
WHtR: Waist-to-height ratio
